# Usability Methods and Attributes Reported in Usability Studies of Mobile Apps for Health Care Education: Protocol for a Scoping Review

**DOI:** 10.2196/19072

**Published:** 2020-08-04

**Authors:** Susanne Grødem Johnson, Thomas Potrebny, Lillebeth Larun, Donna Ciliska, Nina Rydland Olsen

**Affiliations:** 1 Department of Occupational Therapy Faculty of Health and Function Western Norway University of Applied Sciences Bergen Norway; 2 Centre for Evidence-Based Practice Western Norway University of Applied Sciences Bergen Norway; 3 Division of Health Services Norwegian Institute of Public Health Oslo Norway; 4 Faculty of Health Sciences McMaster University Hamilton, ON Canada; 5 Department of Physiotherapy Faculty of Health and Function Western Norway University of Applied Sciences Bergen Norway

**Keywords:** user-computer interface, mobile app, online learning, health education, students

## Abstract

**Background:**

E-learning technologies, including mobile apps, are used to a large extent in health care education. Mobile apps can provide extendable learning environments and motivate students for adaptive and collaborative learning outside the classroom context. Developers should design practical, effective, and easy-to-use mobile apps. Usability testing is an important part of app development in order to understand if apps meet the needs of users.

**Objective:**

The aim of this study is to perform a scoping review of usability methods and attributes reported in usability studies of mobile apps for health care education.

**Methods:**

The scoping review is guided by the methodological framework developed by Arksey & O’Malley and further developed by Levac et al and Kahlil et al. The stages we will follow are as follows: (1) identifying the research question; (2) identifying relevant studies; (3) selecting studies; (4) charting the data; and (5) summarizing and reporting the results. We have developed two research questions to meet the aim of the study, which are as follows: (1) What usability methods are used to evaluate the usability of mobile apps for health care education? and (2) What usability attributes are reported in the usability studies of mobile apps for health care education? We will apply a comprehensive search of the literature, including 10 databases, a reference search, and a search for grey literature. Two review authors will independently screen articles for eligibility.

**Results:**

The initial electronic database searches were completed in March 2019. The literature search identified 14,297 unique references. Following title and abstract screening, the full texts of 369 records were obtained. The scoping review is expected to be completed in spring 2021.

**Conclusions:**

We expect the overview of usability methods and attributes reported in usability studies of mobile apps for health care education to contribute to the knowledge base for researchers and developers. It will give an overview of the research field and provide researchers and developers with relevant and important information on the usability research area, including highlighting possible research gaps.

**International Registered Report Identifier (IRRID):**

DERR1-10.2196/19072

## Introduction

### Background

There has been increasing attention for e-learning technologies, including mobile apps, in health care education. Mobile apps can provide extendable learning environments and motivate students for adaptive and collaborative learning outside the classroom context [1,2]. However, mobile apps have small screen sizes and connectivity problems, and the context provides distractions for the user [3]. Developers of mobile apps need to ensure that apps are practical, effective, and easy to use [1]. Usability testing is important in app development in order to understand how mobile apps meet the needs of users [4]. According to the International Organization for Standardization (ISO), usability is defined as “The extent to which a system, product, or service can be used by specified users to achieve specified goals with effectiveness, efficiency, and satisfaction in a specified context of use” [5].

### Usability Methods

Usability methods, which are currently referred to in usability studies, involve laboratory experiments and field studies [1,6]. There are advantages and disadvantages for both methods. Laboratory experiments take place in a usability laboratory, where the test procedure is conducted in a controlled environment. In a laboratory, researchers can record user activity while they fulfil predefined tasks for later analysis [6], and they can control other irrelevant variables [3]. It is however not possible to test real-world problems (eg, only brief episodes of available time during clinical placement) or problems with internet connection. The expense of instruments and dedicated space make laboratory experiments more costly than other methods [6]. Field studies involve the collection of real-time data from users performing tasks in the real-world environment. In field studies, data about task flows, inefficiencies, and the organizational and physical environments are collected [6]. Field studies allow for data collection within the dynamic nature of the context, which is almost impossible to simulate in a laboratory experiment [1]. However, as users move around in field studies, data collection and conditions are difficult to control [1]. It can also be challenging to collect data in a precise and timely manner [7].

### Usability Attributes

Usability attributes are features used to measure the quality of mobile apps [1]. The three most common usability attributes are effectiveness, efficiency, and satisfaction [3], and all three are part of the ISO standard for usability [5]. Other attributes are learnability, memorability, errors, simplicity, comprehensibility, and learning performance [7]. Selecting appropriate usability attributes depends on the nature of the e-learning technology and the research question of the usability study [7]. It is unclear which usability attributes are most relevant to mobile apps for health care students, although Sandars [8] highlighted the following four main domains for usability testing of e-learning: the learner, technological aspects (navigation, learnability, accessibility, consistency, and visual design), instructional design aspects (interactivity, content and resources, media use, and learning strategy design), and the context.

Previous reviews on usability methods examined usability testing in general [9] or usability specifically related to mobile apps [3,6,7,10]. Only one systematic review specifically explored the usability of mobile learning apps [1], although it did not include studies from health care education. Thus, there is a need for an overview of studies reporting on usability evaluations of mobile apps related to health care education. The aim of this study is to perform a scoping review of usability methods and attributes reported in usability studies of mobile apps for health care education.

## Methods

### Overview

A scoping review summarizes and disseminates research findings to describe the breadth and range of research in a particular topic or field [11-13]. To address the objectives of this scoping review, we will follow the framework for scoping reviews developed by Arksey & O’Malley [11], which was further developed by Levac et al [12] and Kahlil et al [13]. We will adopt the following five stages of this framework: (1) identifying the research question; (2) identifying relevant studies; (3) selecting studies; (4) charting the data; and (5) summarizing and reporting the results [11-13]. A detailed presentation of each step is provided below. This scoping review will also follow the PRISMA-ScR checklist for reporting scoping reviews [14].

#### Stage 1: Identifying the Research Question

Research questions in a scoping review are broad and have a goal to summarize the breadth of the evidence, although the research questions should include a clear scope of inquiry [12]. We have developed two research questions to meet the aim of the study, which are as follows: (1) What usability methods are used to evaluate the usability of mobile apps for health care education? and (2) What usability attributes are reported in usability studies of mobile apps for health care education?

#### Stage 2: Literature Search (Identifying Relevant Studies)

The term usability is defined and used in multiple ways, making it hard to develop a comprehensive search strategy for the term. Using a broader search may be preferable [15]. Therefore, the sensitivity (finding as many relevant articles as possible) of the search is prioritized over the specificity (making sure retrieved articles are relevant), as recommended in order not to miss any relevant articles [16].

We will search the following 10 electronic databases covering technology, education, and health care: Engineering Village (Elsevier), Scopus (Elsevier), ACM Digital Library, IEEE Xplore, Education Resource Information Center (ERIC) (EBSCOhost)*,* PsycINFO (Ovid), CINAHL (EBSCOhost), Medline (Ovid), Embase (Ovid), and Web of Science (Clarivate Analytics). The database searches will be updated before final analysis. The search strategy has been developed in cooperation with a research librarian at Western Norway University of Applied Science. The search string has been peer reviewed by another research librarian, according to the Peer Review of Electronic Search Strategies (PRESS) [17]. A comprehensive search strategy combining text and mesh words relating to health care students and mobile apps was developed. The Boolean operator OR will combine words of similar meaning and the Boolean operator AND will combine searches with words of different meanings. The search strategy for PsycINFO is presented in Multimedia Appendix 1. We will tailor the search strategy to the other databases and present it in our scoping review.

We will browse OpenGrey for grey literature. We will perform a citation search in Google Scholar for included studies and screen reference lists for possible relevant studies. There will be no language restrictions. Studies from January 2008 to the date the searches are run will be sought. The year restriction has been chosen as mobile apps did not appear until 2008 [18].

#### Stage 3: Data Selection (Selecting Studies)

The Rayyan online management software [19] will be used for the selection of eligible studies. Based on the inclusion criteria outlined in Textbox 1, two authors will independently screen the titles and abstracts of studies retrieved from the searches to identify eligible studies We will include research articles of both quantitative and qualitative designs within the area of health care professional education. Commentaries, discussion papers, book editorials, and conference abstracts will be excluded. Moreover, studies relating to learner management systems, e-learning platforms, open online courses, or distance education will be excluded. Studies will be screened in full text, if one reviewer decides to include it. The full text of these potentially eligible studies will be retrieved, imported to the EndNote X9 reference management system [20], and independently assessed for eligibility by two review authors. Any eligibility disagreements will be resolved through discussion or with a third reviewer. A flow chart of the study selection process will be presented.

**Study eligibility.**

**Inclusion criteria**

Population: Studies reporting on health care and allied health care students at the undergraduate and postgraduate levels.

Concepts: Studies of usability testing or usability evaluation methods of mobile apps, where the purpose is related to development of the apps. The usability attributes include effectiveness, efficiency, satisfaction, learnability, memorability, errors, simplicity, comprehensibility, and learning performance of the learning app.

Context: Typical educational settings (eg, classroom teaching, clinical placement, and simulation training).

#### Stage 4: Charting the Data

A standardized prepiloted data extraction form will be used to extract characteristics and data from the included studies. One review author will extract the data from the included studies, which will be checked by another review author. A combination of Microsoft Excel software [21] and NVivo 12 [22] will be used to facilitate this process. Discrepancies will be identified and resolved through discussion or with a third author when necessary.

The process of extracting information from the included studies in a scoping review is an iterative approach [12,13]. This means that we will extract predefined themes, although other relevant information may be included later in the process. Extracted information related to the purpose of the scoping review will include the following:

(1) Study: author(s) name(s), year of publication, title, country, publication journal, study setting, study design, research question, and research methods

(2) Population: number of participants, description of participants, and education level

(3) Concepts: usability methods, usability attributes, modes of delivery, usability phase, materials, procedures, type(s) of location(s), number of usability testing procedures, and modifications

(4) Context: educational setting

#### Stage 5: Summarizing and Reporting the Results

The fifth stage of the scoping review involves summarizing and reporting the results of the included studies [11-13]. The characteristics of each study will be mapped, and a descriptive narrative account will be presented. We will perform a content analysis [23] to map the different usability methods and usability attributes used in the included studies. Tables and graphical illustrations will be used to bring together and present the usability methods and attributes.

### Ethics

This protocol for a scoping review does not require ethical approval or consent to participate. The data consist of data from published articles and do not include individual data.

## Results

**Figure 1:**
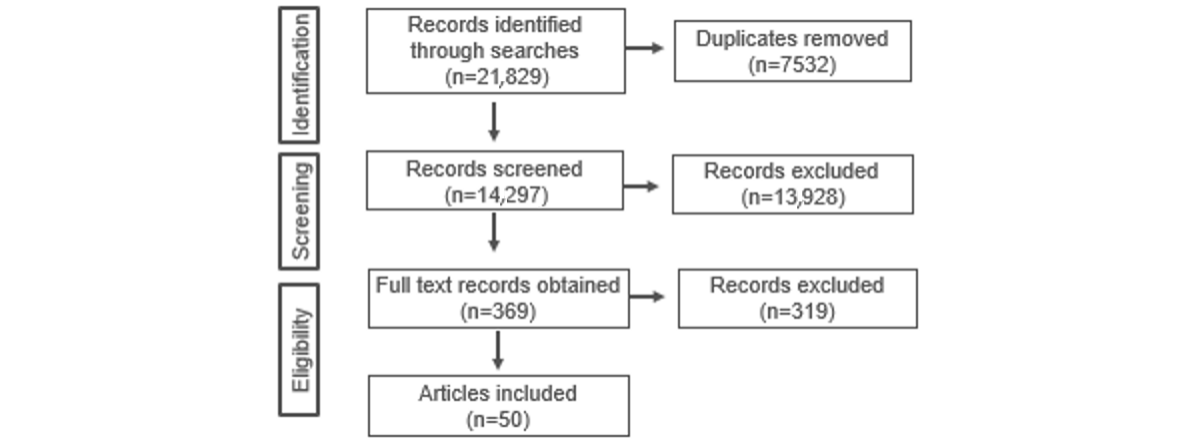
Flow chart of the search results and screening process.

The electronic searches for eight of the databases were completed on March 5, 2019. The literature search identified 14,297 unique references (Figure 1). Owing to the sensitivity of the search, many of these references were irrelevant and excluded. Following title and abstract screening, full texts of 369 records were obtained. Our next step is to assess these references for eligibility.

## Discussion

### Usability Studies of Mobile Apps for Health Care Education

The increasing acceptability and use of mobile apps in the health care education context can lead to improved learning outcomes. However, in order to make learning tools relevant to students, mobile apps must meet the expectations of users [4]. To our knowledge, no overview exists on usability studies of mobile apps for health care education. The results of this scoping review will provide valuable information to developers of mobile apps for health care education, as it will point to relevant usability methods and attributes. Furthermore, the review will identify areas where further research is needed.

A strength of this study is the broad search strategy. We searched ten different databases, and the search strategy was designed in collaboration with a research librarian and was peer reviewed by another research librarian. The search has a time restriction from 2008, but no language restriction. The time restriction was set from 2008, as mobile apps appeared in 2008. A broad search strategy may be associated with lower precision, making it challenging to retrieve relevant articles. We did however experience some challenges with the initial database searches. The authors and research librarians had little experience with databases in academic areas outside health care (eg, Engineering Village and Scopus). “Usability” was not used as a term in the search strategy, as studies on usability do not necessarily refer to or use the term usability. Designing an effective search strategy that balances sensitivity and precision was demanding. Consequently, the search was challenging to narrow, and the search yielded 14,297 unique hits. To ensure that members of the review team had a similar understanding of the inclusion and exclusion criteria, efforts were made to calibrate our screening. Reporting methodological rigor and transparency in a scoping review is of importance to the trustworthiness of the research [24]. Publishing a protocol of the scoping review will support the transparency of the methodology and will assist in the conduction of the scoping review. Following the reporting guidelines for scoping reviews (PRISMA-ScR) [14] will help ensure the methodological quality of the scoping review.

### Conclusion

This scoping review will advance the field of mobile app development for health care education by presenting advice on the relevant usability methods and attributes to study. It will give an overview of the field and provide researchers and developers with relevant and important information on the usability research area, including highlighting possible research gaps.

## Appendix

Multimedia Appendix 1 Search string for PsycINFO.

## Abbreviations

ISO: International Organization of Standardization
