# Supplementary material for: Usability Methods and Attributes Reported in Usability Studies of Mobile Apps for Health Care Education: Protocol for a Scoping Review
Source: JMIR Res Protoc. 2020 Aug 4;9(8):e19072. doi: 10.2196/19072 (PMC7435642; doi:10.2196/19072)
Supplement: Multimedia Appendix 1 [file resprot_v9i8e19072_app1.docx]

**Multimedia Appendix 1: [Search string PsycINFO]**

| **PsycINFO via Ovid – 22.02.2019** | | |
| --- | --- | --- |
| **PsycINFO 1806 to February Week 1 2019 via Ovid** | | |
| **#** | **Searches** |  |
| #1 | students/ or dental students/ or medical students/ |  |
| #2 | ((student* or graduate* or postgraduate* or undergraduate*) adj3 (nurs* or medical or medicine or physiotherap* or physical therap* or occupational therap* or midwife* or social work* or social education or social educator* or medical technolog* or radiography or radiolog* or pharmac* or dentist* or veterinar* or psycholog* or chiropract* or health or healthcare)).ti,ab. |  |
| #3 | #1 or #2 |  |
| #4 | (mobile learning or mlearning or m-learning).ti,ab. |  |
| #5 | (smartphone* or smart phone* or mobile phone* or cell phone* or phone* or telephone*).ti,ab. |  |
| #6 | (tablet* adj3 computer*).ti,ab. |  |
| #7 | (handheld computer* or hand-held computer* or handheld phone* or hand-held phone* or handheld device* or hand-held device* or PDA*).ti,ab. |  |
| #8 | (app or apps or application* or learning app* or application device* or learning application* or web 2*).ti,ab. |  |
| #9 | mobile devices/ or cellular phones/ |  |
| #10 | #4 or #5 or #6 or #7 or #8 or #9 |  |
| #11 | #3 and #10 |  |
| #12 | limit #11 to yr="2008 -Current" |  |
